# Multiple dimensions of health locus of control in a representative population sample: ordinal factor analysis and cross-validation of an existing three and a new four factor model

**DOI:** 10.1186/1471-2288-11-114

**Published:** 2011-08-12

**Authors:** Christiane Otto, Gallus Bischof, Hans-Jürgen Rumpf, Christian Meyer, Ulfert Hapke, Ulrich John

**Affiliations:** 1 University of Göttingen; Department of Medical Psychology & Sociology, Waldweg 37, 37073 Göttingen, Germany; 2 University of Lübeck, Research group S:TEP, Department of Psychiatry and Psychotherapy, Ratzeburger Allee 160, 23538 Lübeck, Germany; 3 University of Greifswald, Institute of Epidemiology and Social Medicine, Walther-Rathenau-Str. 48, 17475 Greifswald, Germany; 4 Robert Koch-Institute, FG 22, Seestr. 10, 13353 Berlin, Germany

## Abstract

**Background:**

Based on the general approach of locus of control, health locus of control (HLOC) concerns control-beliefs due to illness, sickness and health. HLOC research results provide an improved understanding of health related behaviour and patients' compliance in medical care. HLOC research distinguishes between beliefs due to Internality, Externality powerful Others (POs) and Externality Chance. However, evidences for differentiating the POs dimension were found. Previous factor analyses used selected and predominantly clinical samples, while non-clinical studies are rare. The present study is the first analysis of the HLOC structure based on a large representative general population sample providing important information for non-clinical research and public health care.

**Methods:**

The standardised German questionnaire which assesses HLOC was used in a representative adult general population sample for a region in Northern Germany (N = 4,075). Data analyses used ordinal factor analyses in LISREL and Mplus. Alternative theory-driven models with one to four latent variables were compared using confirmatory factor analysis. Fit indices, chi-square difference tests, residuals and factor loadings were considered for model comparison. Exploratory factor analysis was used for further model development. Results were cross-validated splitting the total sample randomly and using the cross-validation index.

**Results:**

A model with four latent variables (Internality, Formal Help, Informal Help and Chance) best represented the HLOC construct (three-dimensional model: normed chi-square = 9.55; RMSEA = 0.066; CFI = 0.931; SRMR = 0.075; four-dimensional model: normed chi-square = 8.65; RMSEA = 0.062; CFI = 0.940; SRMR = 0.071; chi-square difference test: p < 0.001). After excluding one item, the superiority of the four- over the three-dimensional HLOC construct became very obvious (three-dimensional model: normed chi-square = 7.74; RMSEA = 0.059; CFI = 0.950; SRMR = 0.079; four-dimensional model: normed chi-square = 5.75; RMSEA = 0.049; CFI = 0.965; SRMR = 0.065; chi-square difference test: p < 0.001). Results were confirmed by cross-validation. Results based on our large community sample indicated that western general populations separate health-related control-beliefs concerning formal and informal assistance.

**Conclusions:**

Future non-clinical HLOC studies in western cultures should consider four dimensions of HLOC: Internality, Formal Help, Informal Help and Chance. However, the standardised German instrument needs modification. Therefore, confirmation of our results may be useful. Future research should compare HLOC structure between clinical and non-clinical samples as well as cross-culturally.

## Background

Health locus of control (HLOC) is a psychological concept concerning control beliefs in relation to illness, sickness and health. This concept is based on the general approach of locus of control (LOC) developed within the social learning theory by Rotter [1,2]. General LOC is of fundamental importance in psychology, relevant in established approaches concerning depression [3,4] and helplessness [5]. Moreover it is closely connected to the concept of self-efficacy [6]. Due to the assumption that general control beliefs could differ from control beliefs concerning health, the specific construct of HLOC was developed and received increased attention in health research over the last 30 years [7,8]. HLOC research results have been important in understanding health related behaviours, outcomes and care [7]. One main interest of this field of research concerns the compliance of patients in medical care in order to understand patients' adherence to recommended treatments including medication and health related behaviour [9].

In line with the general construct of LOC, HLOC research assumed a three-dimensional construct with control beliefs concerning Internality, Externality powerful Others (POs) and Externality Chance (Chance) [7,8]. HLOC research revealed that patterns of HLOC scores differ for patients with specific diseases [10-12]; furthermore, higher scores on Externality scales seemed to be associated with less education [e.g.[13,14]]. The Multidimensional Health Locus of Control scales (MHLC) [15], parallel Forms A (MHLC-A) and B (MHLC-B), were primarily used in order to assess the three HLOC dimensions [8]. MHLC-A and -B were equivalent, developers reported corresponding correlations for the scales of both forms (for Internality scales r = 0.801, for POs Scales r = 0.761 and for Chance scales r = 0.734) [15].

The MHLC scales were applied to different languages [e.g.[16,17]] and cross-cultural differences in HLOC were investigated. A study which compared Asian women to British Caucasian women found higher scores for the Asian women on both Externality scales in line with the study's expectations [18]. These results show the Asian cultures stronger beliefs in communal values such as the importance of assisting others as well as the belief in fate as compared to more individualistic western cultures. Surprisingly, the Asian women also revealed stronger Internality compared to western women. This difference was the result of a stronger religiosity of the Asian women indicating culturally different interpretations of the MHLC items: Asian women with a strong belief in 'Allah' had simultaneously strong beliefs in their own actions assuming to help themselves by trusting in 'Allah'. However, the authors argued that the structure of HLOC has not been investigated by factor analysis in a corresponding sample, i.e. the HLOC construct may differ structurally over cultures [18].

In western cultures, several factor analyses using mostly selected clinical samples confirmed the three-dimensional structure [e.g.[19-21]]; however, a number of studies failed [e.g.[22,23]]. Another western study analysed a mixed clinical sample (N = 588) and detected a four-dimensional HLOC structure developing and validating Form C of the MHLC (MHLC-C) for condition-specific measuring [24]. The authors confirmed original scales Internality and Chance, but had to differentiate POs scale into one scale concerning doctors and the other concerning family and friends. A latter clinical study examined the Italian version of the MHLC-C in a sample of HIV+ patients (N = 478) via methods based on structural equation modeling (SEM) and showed the superiority of the four- over the three-dimensional construct [25]. However, the four-dimensional construct is not implemented in HLOC research and has not been investigated in a non-clinical sample by now.

To our knowledge, the HLOC construct has never been factor analysed utilising a representative general population sample throughout 30 years of research. Diseases and clinical symptoms concerning a great variety of conditions are given in the general population, but the majority should be healthy. An investigation of the HLOC structure in a western general population sample could provide important results for future research and help to predict and understand the compliance of individuals concerning public health care. In addition, such a study could offer an orientation for non-clinical research. Previous factor analyses using non-clinical samples investigated selected student populations [26-28], the staff of a psychiatric hospital [29] and employees of a university voluntarily participating in a health promotion program [30]. However, a study investigating the general population is lacking.

The aim of the present study was to compare the three- and the four-dimensional construct of HLOC on grounds of a large general population sample representative for a region in Northern Germany by means of ordinal factor analyses based on SEM. We expected to find superiority of the four-dimensional construct and we aimed to confirm our results by cross-validation. Concerning the realisation of our analyses and their presentation we followed guidelines of statistical researchers [31-33]. Our analyses aimed to follow a confirmatory approach comparing theory based models which represented HLOC constructs of differing dimensionality.

## Methods

Data were derived from the project "Transitions in Alcohol Consumption and Smoking" (TACOS). Detailed information about TACOS have already been published [34].

### Sample

A representative sample of the general population of the northern German city of Lübeck and surrounding communities representing the catchment area was used. Individuals aged between 18 and 64 years with residence in the study area were included in the study. To avoid bias due to language problems, German nationality was defined as an additional inclusion criterion. Participants were randomly selected from the registration office files of all 47 communities representing the study area. The response rate was 70.2% and the final sample consisted of 4,075 individuals.

The study followed the ethical principles of the American Psychological Association [35]. Individuals gave written informed consent and were informed that they were free to participate and could withdraw from the study at any time. At the time of data assessment (June 1996 to March 1997), it was not mandatory at the University of Lübeck to consult the ethical committee. However, our proceeding was in line with the Helsinki Declaration [36].

### Assessment

The German modification of the MHLC, i.e. the questionnaire to survey control beliefs concerning disease and health was used (KKG) [37]. The KKG was the recommended instrument to be used in German speaking samples to assess HLOC [38]. Two alternative German instruments were available, but the questionnaire named "health related control beliefs" was a short-form offering only a total of nine items (GKÜ) [39], while the questionnaire to assess health related control beliefs assumed a bipolar construct of HLOC (Externality versus Internality; FEGK) [40]. However, the KKG assumed the original three HLOC dimensions Internality, POs and Chance with seven items per scale.

The KKG is theoretically in line with the MHLC-A and -B [38]. Additionally, both instruments, MHLC-A/-B as well as the KKG, were developed based on non-clinical data [15,37,41]. MHLC-A and -B could be used for respondents of at least 16 years of age, while the KKG was also appropriate for younger individuals (≥ 12 years of age). Furthermore, KKG items were phrased focussing on 'sickness' (literally translated: 'physical complaints'), while MHLC-A/-B developers used words like 'health' and 'illness'. Developers of MHLC-A/-B recruited respondents at an airport (age ≥ 16 years; n = 115) offering a pool of 81 items. Items for the MHLC-A/-B were selected respecting several item criteria (i.e. mean close to the midpoint of the answering scale, wide distribution of responses, significant correlation to a priori scale, low correlation to measure of social desirability and wording). Items for the KKG were selected out of a pool of 35 items based on principal components analysis (PCA) with respect to corrected item scale correlations and retest reliabilities (n = 122; pupils ≥ 12 years of age and students). The three-dimensional structure of the KKG has been confirmed by another PCA based on a second non-clinical sample (n = 366; pupils ≥ 12 years of age and adults) [37,41]. Further studies confirmed the validity of the KKG due to associations to external variables [42-44] and a number of studies have been published using this questionnaire [e.g. [45-47]].

We assumed the KKG may also serve to assess four dimensions of HLOC corresponding to the MHLC-C. POs scale included three items concerning Formal Help (# 02, 10, 12) and four items regarding Informal Help (# 04, 06, 14, 20). Formal Help dimension corresponded to the scale concerning doctors and Informal Help corresponded to the scale concerning family and friends of the MHLC-C [24]. In the present study, items were offered with five point Likert scales ("strongly disagree" to "strongly agree") and the KKG was used in standardised German form. However, Chance scale item 11 was changed with respect to double negation effects [48,49]. The original phrasing of item 11 ("If I am feeling well or not, cannot be affected") was modified ("If I am feeling well or not, can be affected"). Table 1 presents KKG items.

**Table 1:** KKG^1 ^items translated into English and checked by back-translation

Items	item wordings
**01**	When I feel bad physically then this is my own fault.

**02**	When I have a medical condition, I usually go to the doctor.

**03**	Whether or not my sickness continues depends on Chance.

**04**	When I feel well physically I owe this to all the advice and help of others.

**05**	When I get sick then I didn't take sufficient care of myself.

**06**	When I have a sickness I seek out the advice of others.

**07**	Bodily sickness cannot be influenced: When I am unlucky it appears suddenly.

**08**	When I take care of myself I have no sickness.

**09**	When destiny wants it I experience bodily pain.

**10**	When I am sick I ask an expert to help me.

**11^2^**	It can be influenced whether or not I feel well.

**12**	When I don't have a good doctor I more often suffer from sickness.

**13**	Whether or not sickness disappears depends on whether or not I'm lucky.

**14**	I can avoid sickness when I take the advice of others.

**15**	I owe it to my Fate when my sickness disappears.

**16**	When I know enough about myself I can help myself when I am sick.

**17**	When I am sick I know that I can help myself.

**18**	It is up to me whether my sickness abates.

**19**	I believe that Luck and Chance play a big role in my physical health.

**20**	When I feel bad others know better than me how I feel.

**21**	It's up to me to protect myself from sickness.

^1 ^German questionnaire to survey control beliefs concerning disease and health by Lohaus and Schmidt (1989) [37]; ^2 ^item 11 was modified in the present study.

### Data analyses

#### Software

Interactive LISREL 8.80 [50] was used for SEM based analyses. Additionally, SPSS 14.0 [51] served for calculation of Spearman correlations between item 11 and original HLOC scales. Furthermore, Mplus 5.21 [52] was exclusively used to calculate specific chi-square difference tests not offered by LISREL.

#### Preliminary analyses and data generation

The total sample was randomly split, subsample A served for model development (i.e. initial model comparison and further model development) and subsample B for validation. To present our ordinal raw data we calculated univariate frequency distributions of KKG items based on subsample A. Data were treated as ordinal calculating polychoric correlation and asymptotic covariance matrices and using robust diagonal weighted least squares (robust DWLS) estimation method [32,53]. The use of polychoric correlations required underlying bivariate normality for each pair of items [32]. With respect to the rephrasing of item 11, scale allocation of this item had to be determined calculating Spearman correlations to original HLOC scales by SPSS.

#### Initial model comparison

A sequence of theory-driven nested models was specified starting with a very parsimonious model [33]. HLOC dimensions are represented by latent variables in the models with variances fixed to one as recommended [32,33]. Each item (i.e. indicator) was allocated to one and only one latent variable within each specified model. Model 1 had one general latent variable, while Model 2 included two latent variables corresponding to dimensions Internality and Externality (i.e. original dimensions POs and Chance combined) [54]. Model 3 represented the original construct with dimensions Internality, POs and Chance [8,15]. Finally, Model 4 represented the four-dimensional construct differentiating original POs dimension into Formal Help and Informal Help. We assumed correlated, but independent latent variables within our models corresponding to former research and with respect to varying correlations among HLOC scales reported in different studies [e.g. [15,29]]. In line with this proceeding, statistical researchers have recommended allowing correlations among latent variables in SEM based confirmatory factor analyses due to methodological differences compared to exploratory methods [55].

Models were tested by confirmatory factor analysis (CFA). Satorra Bentler scaled chi-square (SB-scaled chi-square) [56] correcting for non-normality was used for our ordinal data [32]. In order to respect the complexity of our models, we calculated the normed chi-square index by dividing the SB-scaled chi-square value for each model by its degrees of freedom [57,58]. According to known biases of chi-square statistics depending on sample size, that also affect the normed chi-square index, descriptive fit indices were considered [e.g.[59]]. The frequently used root mean square error of approximation (RMSEA) [60] accompanied by its associated 90% confidence interval (CI) and a p-value due to a close fit test (i.e. RMSEA < 0.05) were calculated. Additionally, the standardised root mean square residual (SRMR) [61] and the incremental comparative fit index (CFI) [62] were chosen. Selected indices are robust towards sample size effects [e.g.[59]]. SRMR and CFI are additionally recommended for analyses using asymptotic distribution free estimation methods as used in the present analyses [63].

For inferential model comparison chi-square difference tests are highly appreciated [59]. Note that the use of chi-square difference tests is more appropriate than the use of chi-square tests [33]. However, no Monte Carlo studies were found indicating which specific difference test would be appropriate to use in ordinal data specific analyses in LISREL. There may be a need for further developments in statistical research and theory concerning this question. In the present analyses, these tests were calculated with the alternative software Mplus using the weighted least squares means and variance adjusted estimation method (WLSMV) [64]. Robust DWLS offered by LISREL and WLSMV offered by Mplus are very similar; both procedures respect ordinal data by investigating polychoric correlations and asymptotic covariances using weighted least squares estimation methods. Concerning results of chi-square difference tests, we only interpreted resulting p-values as recommended [65]. For additional model comparison residuals and factor loadings were respected [e.g.[66]].

#### Further model development

Based on the recommendations of statisticians, we aimed to decide about the number of factors using SEM based CFA in our analysis of a construct that provided a solid theoretical basis [67]. However, CFA results and residual analyses indicated some problems concerning the performance of specific KKG items. Therefore, exploratory factor analysis (EFA) was used to evaluate the performances of KKG items in solutions corresponding to our CFA models. LISREL offers MINRES and ULS exploratory factor analyses both useable for ordinal data. Results of both methods are very similar [68]. With respect to our ordinal data, polychoric correlation matrices were analysed by MINRES [69] and factor loadings gained by promax rotation were considered in order to assume consistently correlated but independent factors in our study. MINRES provides factor loadings for unrotated, varimax- rotated and promax rotated solutions, while rotated solutions are transformations of the unrotated solution [68]. Additionally, minimum fit function chi-square (CMin) was calculated. Furthermore, a reference variable solution using instrumental variable methods offered t-values for factor loadings [68].

#### Model validation

The model specific two-sample cross-validation index (CVI) [70,71] was used. CVI served to cross-validate our results of model comparison. Model comparison and further model development were conducted based on subsample A, results were cross-validated respecting subsamples A and B. The CVI indicated the discrepancy between the model-implied data matrix of the calibration sample (i.e. subsample A) and the empirical data matrix of the validation sample (i.e. subsample B). Due to fact that the two-sample CVI respected both data sets this index directly indicated which model cross-validated best [70,71]. Therefore, the CVI served to check for "capitalisation on chance" [70,71]. That is, fluctuations caused by random may appear in subsample A, while in subsample B different chance fluctuations were likely even though both samples were from the same population.

This index had to be used within a sequence of nested models respecting the so-called saturated model. The saturated model was an abstract model that fit the data perfectly, i.e. model implied and empirical data matrices were identical indicated by a chi-square of 0.0 [33]. In the present study, ordinal data specific CVI was calculated [72].

## Results

### Preliminary item analyses and data generation

After randomly splitting the total sample, subsample A was analysed (n = 2,037). Univariate frequency distributions and missing answers for KKG items in subsample A are presented in Table 2.

**Table 2:** Univariate frequency distributions of health locus of control items in subsample A

Items	Strongly disagree (1)	2		3		4	strongly agree (5)	*missing answers*
**01**	487	354		614		362	213	*7*

**02**	270	404		487		371	498	*7*

**03**	1079	343		378		124	97	*16*

**04**	989	439		358		143	101	*7*

**05**	459	412		586		358	209	*13*

**06**	457	416		555		398	200	*11*

**07**	549	389		451		310	327	*11*

**08**	395	379		671		380	203	*9*

**09**	922	293		427		202	181	*12*

**10**	140	241		489		542	619	*6*

**11**	288	209		573		544	417	*6*

**12**	987	345		383		142	173	*7*

**13**	1122	370		332		118	85	*10*

**14**	566	342		599		360	162	*8*

**15**	1086	345		342		119	137	*8*

**16**	167	195		635		624	404	*12*

**17**	199	293		761		490	284	*10*

**18**	282	382		757		401	202	*13*

**19**	729	419		453		243	183	*10*

**20**	1167	415		282		100	66	*7*

**21**	162	209		657		543	459	*7*

Listwise deletion was used due to a small number of cases with missing values (n = 69; 3.39%). Spearman correlation of item 11 to corrected original Chance scale was very low (r = 0.115; p < 0.001), while a remarkable correlation to original Internality scale occurred (r = 0.398; p < 0.001). Therefore, item 11 was allocated to dimension Internality in the following analyses. The condition of bivariate normality was not fulfilled for item pair 16 and 17 (specific RMSEA = 0.114; p = 0.01). An item sequence effect could only be considered for the latter item; thus item 17 was excluded. Subsequently, data matrices were calculated. Polychoric correlations of subsample A are presented in an additional file, we added polychoric correlations of subsample B into this file (see Additional file 1: Polychoric correlations of health locus of control items using subsamples A and B).

### Initial model comparison

Results of model testing are presented in Table 3. Model-testing procedure revealed large chi-squares for all models due to our large sample size as expected. Concerning descriptive fit indices, we respected the following "rules of thumb": A normed chi-square < 5 indicated an adequate model fit, while a value ≥ 2 denoted a close fit [33]. An RMSEA ≤ 0.05 indicated a close fit, while values up to 0.08 denoted adequate fits [73]. Similarly, an SRMR < 0.05 suggested a close fit and a value up to 0.10 represented an adequate fit [74]. A CFI > 0.97 denoted a close fit, an adequate fit was indicated by a CFI > 0.95 [75].

**Table 3:** Fit values of analysed models representing constructs of health locus of control

	Model 1^1^	Model 2^2^	Model 3^3^	Model 4^4^
**SB-scaled chi-square^5^**	6,399	3,154	1,595	1,419

p-value	< 0.001	< 0.001	< 0.001	< 0.001

**Degrees of freedom**	170	169	167	164

**Normed chi-square^6^**	37.64	18.66	9.55	8.65

**RMSEA^7^**	0.136	0.095	0.066	0.062

Confidence interval (90%)	0.134-0.139	0.092-0.098	0.063-0.069	0.059-0.065

p-value (RMSEA < 0.05)	< 0.001	< 0.001	< 0.001	< 0.001

**CFI^8^**	0.701	0.857	0.931	0.940

**SRMR^9^**	0.137	0.096	0.075	0.071

^1 ^Model with one general latent variable;

^2 ^Model with latent variables Internality and Externality;

^3 ^Model with latent variables Internality, Externality powerful Others and Chance;

^4 ^Model with latent variables Internality, Formal Help, Informal Help and Chance;

^5 ^Satorra Bentler scaled chi-square developed by Satorra and Bentler (1994) [56];

^6 ^Ratio of SB-scaled chi-square and degrees of freedom proposed by Jöreskog (1969) [57];

^7 ^Root mean square error of approximation developed by Steiger (1990) [60];

^8 ^Comparative fit index developed by Bentler (1990) [62];

^9 ^Standardised root mean residual developed by Bentler (1995) [61].

In the present analyses descriptive fit indices consistently indicated poor fits for Models 1 and 2 (see Table 3). Concerning Models 3 and 4, RMSEAs and SRMRs indicated adequate fits, while normed chi-squares and CFIs were inadequate in both cases.

Concerning Models 1 to 3, all indices indicated obvious and consistent improvements in fit. Correspondingly, chi-square difference tests revealed significant differences between Models 1 and 2 as well as between Models 2 and 3 (p < 0.001 for both comparisons). For Models 3 and 4, differences in indices were smaller. Normed chi-squares, RMSEAs, CFIs and SRMRs indicated improvement in fit, but CIs of RMSEAs for both models overlapped. However, the point estimate of the RMSEA for each model was lying outside the CI of the corresponding other model and chi-square difference test indicated a significant difference between both models favouring Model 4 (p < 0.001).

However, this evaluation of model fit was based on "rules of thumb". These guidelines should not be overgeneralised; instead, fit indices should be used to identify differences in model specifications conducting a comparison of nested models as realised in the present study [76]. In order to analyse our best performing nested models more detailed residuals and factor loadings were investigated.

Model 4 also performed better than Model 3 due to residuals and factor loadings: We detected a very large outlying standardised residual indicating a specification error in Model 3 in the sense of a clear underestimation of the relationship between POs items 02 and 10 (ε_0210 _= 24.7) [66]. These two items additionally revealed low factor loadings in Model 3 (see Table 4). Note that secondary loadings were fixed to zero in CFA models.

**Table 4:** Standardised factor loadings resulting from confirmatory factor analyses

	**Model 3**			**Model 4**			
	**Internality**	**POs^1^**	**Chance^2^**	**Internality**	**Formal** **Help**	**Informal** ** Help**	**Chance**

**Item 01**	0.543			0.548			

**Item 05**	0.652			0.654			

**Item 08**	0.584			0.585			

**Item 11**	0.496			0.493			

**Item 16**	0.561			0.562			

**Item 18**	0.685			0.682			

**Item 21**	0.597			0.595			

**Item 02**		0.360			0.655		

**Item 04**		0.659				0.662	

**Item 06**		0.622				0.631	

**Item 10**		0.317			0.564		

**Item 12**		0.498			0.619		

**Item 14**		0.645				0.659	

**Item 20**		0.675				0.674	

**Item 03**			0.586				0.586

**Item 07**			0.468				0.467

**Item 09**			0.699				0.698

**Item 13**			0.833				0.833

**Item 15**			0.822				0.823

**Item 19**			0.675				0.676

^1 ^Externality powerful Others; ^2 ^Externality Chance.

On the other hand, Model 4 revealed satisfactory factor loadings for all indicators and no outlying large residual. However, no outlying but large negative residuals occurred in this model concerning item 12 (ε_0212 _= -8.7; ε_1012 _= -8.1). This item referred to unpleasant states due to the absence of a good physician, whereas remaining POs items corresponded to the assistance of others (see Table 1). KKG developers had already reported inadequate values for this item within their three factorial solution (loading = 0.30; correlation to remaining POs scale = 0.19) [37,41].

### Further model development

The three-factorial EFA solution provided a normed CMin of 15.74 (CMin = 2094; df = 133), the corresponding value for the four-factorial solution was at 9.99 (CMin = 1159; df = 116) indicating a better fit of the four-factorial solution. Scale scores were means over the items of each scale [37,41] ranging from 1 to 5 for each scale (Internality: mean = 3.10; standard deviation (SD) = 0.79; POs: mean = 2.57; SD = 0.73; Chance: mean = 2.18; SD = 0.87; Formal Help: mean = 2.97; SD = 0.94; Informal Help: mean = 2.27; SD = 0.86).

EFAs confirmed Internality and Chance scales in both solutions with adequate loadings on factors indicated by theory (see Table 5). Concerning POs scale in the three-factorial solution, item 12 loaded relatively low and revealed a relatively high cross loading on the factor representing Chance. Item 20 also showed a high cross loading on the Chance factor, but a solid loading on the POs factor. In the four-factorial solution, factor loadings were highest on theoretically indicated factors for each item, but item 12. This item was allocated to Formal Help scale, but loaded higher on factors representing Informal Help and Chance. The level of these unexpected loadings was inadequate. Statisticians suggested that factor loadings above 0.31 are adequate as long as no high cross loadings appear [77]. The cross loading of item 12 in the three-factorial solution was relatively high; however, first and foremost, the loadings of this item within the four-factorial solution were not in line with the theory. The reference variable solution revealed significant t-values for most factor loadings due to our large sample size (i.e. t-values ≥ |1.96|, not presented). However, the highest t-value for a loading of an item due to the reference variable solution was corresponding to the highest factor loading for this item provided by the promax solution.

**Table 5:** Standardised factor loadings resulting from exploratory factor analyses

	**Three-factorial solution**			**Four-factorial solution**			
	**Factor** **1**	**factor** **2**	**factor** **3**	**Factor** **1**	**factor** **2**	**factor** **3**	**factor** **4**

**Item 01**	**0.520**	0.028	-0.006	**0.455**	0.145	-0.117	-0.048

**Item 05**	**0.572**	0.067	0.029	**0.508**	0.182	-0.090	-0.015

**Item 08**	**0.553**	0.062	-0.024	**0.497**	0.155	-0.081	-0.059

**Item 11**	**0.493**	0.074	-0.071	**0.507**	0.018	0.048	-0.044

**Item 16**	**0.575**	-0.049	0.068	**0.638**	-0.147	0.072	0.127

**Item 18**	**0.690**	-0.065	0.104	**0.712**	-0.078	0.001	0.129

**Item 21**	**0.647**	0.025	-0.122	**0.707**	-0.097	0.117	-0.062

**Item 02**	-0.198	**0.595**	-0.133	-0.103	**0.254**	**0.623**	-0.061

**Item 04**	0.056	**0.553**	0.140	-0.074	**0.738**	-0.035	0.013

**Item 06**	0.087	**0.696**	-0.053	0.043	**0.620**	0.173	-0.083

**Item 10**	-0.097	**0.486**	-0.143	0.095	-0.013	**0.840**	0.003

**Item 12**	-0.044	**0.323**	**0.267**	-0.033	**0.264**	0.157	**0.259**

**Item 14**	0.239	**0.545**	0.039	0.197	**0.516**	0.103	0.004

**Item 20**	0.025	**0.439**	**0.302**	-0.074	**0.592**	-0.040	0.198

**Item 03**	0.022	0.035	**0.564**	-0.058	**0.262**	-0.206	**0.471**

**Item 07**	-0.128	-0.025	**0.490**	-0.044	-0.137	0.149	**0.543**

**Item 09**	-0.081	0.037	**0.674**	-0.013	-0.037	0.116	**0.707**

**Item 13**	0.043	0.042	**0.797**	0.022	0.170	-0.084	**0.737**

**Item 15**	-0.015	-0.019	**0.837**	0.021	0.009	0.007	**0.830**

**Item 19**	0.093	-0.110	**0.724**	0.119	-0.050	-0.051	**0.710**

Loadings ≥ 0.25 are printed in bold.

As we excluded item 12 and recalculated EFAs, loadings of remaining items stayed satisfactory with highest loadings equal to or above 0.45 on factors indicated by theory in both solutions. The normed CMin was at 17.09 for the three-factorial solution (CMin = 1999; df = 117) and at 10.72 for the four-factorial solution (CMin = 1083; df = 101) indicating that the four-factorial solution revealed a better fit. Ranges of scores for POs scale, Formal Help and Informal Help scales remained stable (POs without item 12: mean = 2.65; SD = 0.76; Formal Help without item 12: mean = 3.41; SD = 1.12). Further EFAs forcing up to nine factors revealed no other solution which was theoretically acceptable and therefore worth further examinations.

Three- and four-dimensional CFA models without item 12 were specified (i.e. Models 3a and 4a). Model 3a revealed an adequate fit due to the RMSEA, CFI and SRMR, but the normed chi-square was not adequate (SB-scaled chi-square = 1,154; df = 149; normed chi-square = 7.74; RMSEA = 0.059; CI(90%) = 0.055-0.062; p_(RMSEA < 0.05) _< 0.001; CFI = 0.950; SRMR = 0.079). The fit of Model 4a was also adequate, but closer due to normed chi-square, RMSEA, CFI and SRMR (SB-scaled chi-square = 840; df = 146; normed chi-square = 5.75; RMSEA = 0.049; CI(90%) = 0.046-0.052; p_(RMSEA < 0.05) _= 0.670; CFI = 0.965; SRMR = 0.065). However, normed chi-square was better, but still not fully satisfactory. Chi-square difference test indicated a significant difference in favour of Model 4a (p < 0.001). Furthermore, Model 4a had the most satisfactory residual pattern of any model within this analysis [66].

The latent variable Formal Help of Model 4a explained only two indicators. Therefore, assessment of this dimension was rather unstable. However, methodologists suggested a number of two indicators per latent variable would be appropriate in CFAs based on sample sizes of more than 400 cases [[59]; based on [78,79]]. "There seems to be a mutual compensatory effect of sample size and number of indicators per factor: More indicators may compensate for small sample size, and a larger sample size may compensate for few indicators." [59, page 50, lines 27 to 30].

**Figure 1:**
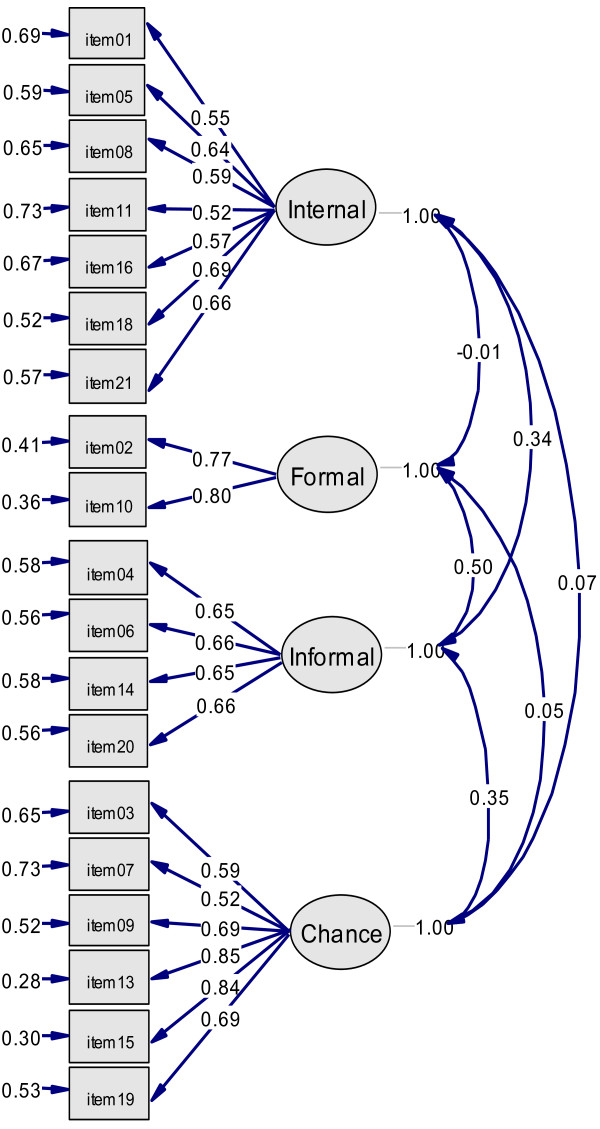
**Four-dimensional model representing health locus of control**. Model 4a included latent variables Internality (Internal), Formal Help (Formal), Informal Help (Informal) and Externality Chance (Chance). Correlations among latent variables, factor loadings and measurement errors of indicators were calculated, while variances of latent variables were fixed to one. Note that items 12 and 17 of the German questionnaire to survey control beliefs concerning disease and health (KKG) by Lohaus and Schmidt (1989) [37] were not included in Model 4a.

Figure 1 presents Model 4a with satisfactory factor loadings and moderate inter-correlations among latent variables.

### Model validation

CVI confirmed consistent improvements in fit from Models 1 to 4 (see Table 6). However, Models 3a and 4a were not nested with Models 1 to 4 due to the use of different data bases [e.g.[59]]. Consequently, Models 3a and 4a could not be directly compared to Models 1 to 4. Though, Models 3a and 4a were nested with each other and the CVI was calculated for the corresponding second sequence of nested models (added in Table 6). CVI confirmed that Model 4a performed better than Model 3a.

**Table 6:** Results of cross-validation for two sequences of nested models

**1^st ^sequence**	Model 1^1^	Model 2^2^	Model 3^3^	Model 4^4^	Saturated Model^5^
**CVI^6^**	3.99	2.53	1.65	1.46	0.45

**2^nd ^sequence**			**Model 3a^7^**	**Model 4a^8^**	**Saturated** **Model a^9^**

**CVI**			1.48	1.11	0.41

^1 ^Model with one general latent variable;

^2 ^Model with latent variables Internality and Externality;

^3 ^Model with latent variables Internality, Externality powerful Others and Chance;

^4 ^Model with latent variables Internality, Formal Help, Informal Help and Chance;

^5 ^Model with perfect fit;

^6 ^Cross-validation index developed by Cudeck and Browne (1983) [71];

^7 ^Model 3a corresponded, generally, to Model 3, but did not include item 12;

^8 ^Model 4a corresponded, generally, to Model 4, but did not include item 12;

^9 ^Model with perfect fit that did not include item 12.

## Discussion

The aim of this study was to analyse the factor structure of HLOC based on the German modification of the MHLC with up-to-date methods in a representative large western general population sample. The model which represented the four-dimensional HLOC construct performed best within our study based on results of initial CFAs. This model was superior to the model which represented the original three-dimensional HLOC construct according to fit indices, chi-square difference tests, residuals and factor loadings. After exclusion of one item due to residual analyses and EFA results, the difference between the models became more obvious. Results of model comparison were confirmed by cross-validation.

Our analyses followed recommendations of statistical researchers [67]. The authors of the aforementioned study advised using SEM based CFA in order to decide about the number of factors of a construct that provides a solid theory. However, the decision to exclude item 12 was based on exploratory methods in the present study. Therefore, CFA and cross-validation were used to confirm these findings.

The present study used the German modification of the MHLC. Even though MHLC-A/-B and the KKG differ in some aspects (i.e. six versus seven items per original scale, suitable minimum age of respondents and phrasing of the items), both instruments focus on the same construct of HLOC [15,37,38,41]. However, our analyses of a large general population sample pointed out weaknesses of the standardised German instrument. We could avoid double negotiation effects by rephrasing item 11 and we had to exclude item 17 due to an item sequence effect caused by repetitive items 16 and 17. Additionally, our study indicated that a rephrasing of item 12 may be needed. On the other hand, we found superiority for the four- over the three-dimensional models with and without item 12 indicated by chi-square difference tests. Methodologists recommended the use of chi-square difference tests for alternative model comparisons and pointed out shortcomings of alternative strategies [59,80]. Concerning the KKG, we assumed that previous studies had not reported as much critique due to the fact that they analysed selected and smaller samples, did not investigate the KKG as detailed, and/or did not differ between Formal Help and Informal Help [e.g.[41,42,45,46]]. However, after excluding item 12, the latent variable Formal Help in our study suffered from instability due to the fact that this dimension was assessed by only two items which were very similar. Even though this proceeding was acceptable due to the methods we used [59], Formal Help dimension needs a broader assessment.

HLOC research has already pointed out the superiority of the four-dimensional construct in clinical samples [24,25]. However, our analysis is the first to compare the three- and four-dimensional HLOC constructs in a representative general population sample of a western culture. Furthermore, previous factor analyses of clinical data focused on the condition-specific construct of HLOC due to the MHLC-C, while we examined the general construct of HLOC. Our results are in line with findings of previous studies investigating the three-dimensional construct. A very early study had already postulated an empirical distinctiveness of professional and non-professional help due to an inadequate performance of item 7 of the MHLC-A in their clinical sample [81]. This item is the only item of this form referring directly to the family. Besides other clinical studies [e.g.[8,22]], non-clinical studies had also reported inadequate values for this item [28,30]. However, results of factor analyses are generally sample specific. In the present study, the representativeness and size of our sample lowered the risk of sample selection bias. Additionally, cross-validation was conducted. We recommend assuming four HLOC dimensions in future non-clinical research in western cultures.

## Conclusions

Our study found evidence indicating that future non-clinical HLOC research in western populations should consider four dimensions of HLOC, i.e. Internality, Formal Help, Informal Help and Chance in order to investigate these beliefs appropriately. Otherwise important information may be missed. Health behaviour and most importantly the compliance of individuals concerning public health care (e.g. in medical care or in health promotion programs) may be better and more appropriately predicted by health related control beliefs concerning medical professionals than by attributions concerning the family and friends, or a mixture of both. However, the standardised German questionnaire to assess HLOC needs modification. Therefore, future research should confirm our findings providing a more stable and broader assessment of the four HLOC dimensions.

Future HLOC research should compare the HLOC structure in clinical and non-clinical samples to analyse and understand this construct in more detail. Only two corresponding multi-group analyses have been published, but both studies assumed the three-dimensional construct and compared individuals suffering from diabetes to healthy controls [54,82]. One study detected differences concerning the interpretation of Internality items between patients with diabetes and healthy controls [54], while the other study found no differences focussing on elderly respondents [82]. Multi-group analyses respecting further diseases are needed. Multi-group analyses could also provide interesting information concerning measurement invariances of the HLOC construct over cultures. Such analyses could investigate the equivalence of the number of factors, factor loadings and factor correlations over samples and may reveal important findings.

## Competing interests

The authors declare that they have no competing interests.

## Authors' contributions

UJ (project manager), CM, HJR, and UH were responsible for design and realisation of the project TACOS including data assessment. GB participated substantially in drafting and editing the present manuscript. CO conducted the statistical analyses for the present study and wrote the manuscript as primary author. All authors read and approved the manuscript.

## Authors' information

PD Dr. phil. H-JR and Dr. phil. GB focus on research concerning early interventions for substance use disorders as scientists at the University of Lübeck, Germany. Prof. Dr. UJ and PD Dr. phil. CM concentrate on research of prevention and epidemiology as scientist at the University of Greifswald, Germany. Dr. UH is psychologist at the Robert-Koch-Institute in Berlin, Germany. Postgraduate psychologist CO is working as a scientist at the University of Göttingen, Germany. The actual paper presents main results of her PhD thesis.

## Pre-publication history

The pre-publication history for this paper can be accessed here:

http://www.biomedcentral.com/1471-2288/11/114/prepub

## Supplementary Material

Additional file 1 **Polychoric correlations of health locus of control items using subsamples A and B**. Polychoric correlations are presented for subsample A (n = 2,037; below diagonal) and for subsample B (n = 2,038; above diagonal). Subsamples A and B were gained by randomly splitting the total sample. Click here for file
