# Supplementary material for: Multiple dimensions of health locus of control in a representative population sample: ordinal factor analysis and cross-validation of an existing three and a new four factor model
Source: BMC Med Res Methodol. 2011 Aug 12;11:114. doi: 10.1186/1471-2288-11-114 (PMC3175204; doi:10.1186/1471-2288-11-114)
Supplement: Additional file 1 — Polychoric correlations of health locus of control items using subsamples A and B. Polychoric correlations are presented for subsample A (n = 2,037; below diagonal) and for subsample B (n = 2,038; above diagonal). Subsamples A and B were gained by randomly splitting the total sample. [file 1471-2288-11-114-S1.PDF]

Items	01	02	03	04	05	06	07	08	09	10	11	12	13	14	15	16	18	19	20	21
**01**		-0.062	0.094	0.131	0.487	0.145	-0.070	0.316	0.060	-0.041	0.263	0.025	0.097	0.147	0.013	0.175	0.345	0.038	0.109	0.303
**02**	-0.062		-0.036	0.219	-0.017	0.314	0.071	0.018	0.075	0.575	-0.026	0.243	0.066	0.163	0.057	-0.079	-0.049	-0.023	0.196	-0.019
**03**	0.094	-0.036		0.335	0.135	0.122	0.296	0.086	0.377	-0.092	-0.018	0.251	0.506	0.185	0.431	0.068	0.081	0.372	0.306	-0.064
**04**	0.131	0.219	0.335		0.217	0.451	0.121	0.157	0.163	0.119	0.079	0.269	0.305	0.400	0.245	0.070	0.099	0.173	0.471	0.025
**05**	0.487	-0.017	0.135	0.217		0.172	-0.002	0.448	0.038	-0.007	0.267	0.063	0.128	0.160	0.095	0.255	0.359	0.064	0.152	0.348
**06**	0.145	0.314	0.122	0.451	0.172		0.049	0.145	0.166	0.239	0.152	0.210	0.185	0.501	0.164	0.118	0.091	0.079	0.360	0.136
**07**	-0.070	0.071	0.296	0.121	-0.002	0.049		-0.068	0.489	0.117	-0.026	0.175	0.322	0.000	0.398	0.079	-0.031	0.277	0.105	-0.069
**08**	0.316	0.018	0.086	0.157	0.448	0.145	-0.068		-0.001	-0.030	0.264	0.042	0.095	0.154	0.023	0.282	0.376	0.057	0.153	0.337
**09**	0.060	0.075	0.377	0.163	0.038	0.166	0.489	-0.001		0.118	-0.004	0.295	0.508	0.149	0.589	0.043	0.039	0.461	0.279	-0.044
**10**	-0.041	0.575	-0.092	0.119	-0.007	0.239	0.117	-0.030	0.118		0.052	0.183	-0.006	0.192	0.038	0.056	0.008	-0.018	0.086	0.098
**11**	0.263	-0.026	-0.018	0.079	0.267	0.152	-0.026	0.264	-0.004	0.052		0.089	0.056	0.241	-0.019	0.353	0.312	0.044	0.017	0.335
**12**	0.025	0.243	0.251	0.269	0.063	0.210	0.175	0.042	0.295	0.183	0.089		0.322	0.259	0.281	0.004	0.096	0.226	0.285	-0.027
**13**	0.097	0.066	0.506	0.305	0.128	0.185	0.322	0.095	0.508	-0.006	0.056	0.322		0.260	0.682	0.099	0.146	0.590	0.407	-0.019
**14**	0.147	0.163	0.185	0.400	0.160	0.501	0.000	0.154	0.149	0.192	0.241	0.259	0.260		0.184	0.243	0.237	0.165	0.377	0.238
**15**	0.013	0.057	0.431	0.245	0.095	0.164	0.398	0.023	0.589	0.038	-0.019	0.281	0.682	0.184		0.115	0.146	0.582	0.361	-0.037
**16**	0.175	-0.079	0.068	0.070	0.255	0.118	0.079	0.282	0.043	0.056	0.353	0.004	0.099	0.243	0.115		0.476	0.126	0.011	0.408
**18**	0.345	-0.049	0.081	0.099	0.359	0.091	-0.031	0.376	0.039	0.008	0.312	0.096	0.146	0.237	0.146	0.476		0.194	0.114	0.456
**19**	0.038	-0.023	0.372	0.173	0.064	0.079	0.277	0.057	0.461	-0.018	0.044	0.226	0.590	0.165	0.582	0.126	0.194		0.302	0.071
**20**	0.109	0.196	0.306	0.471	0.152	0.360	0.105	0.153	0.279	0.086	0.017	0.285	0.407	0.377	0.361	0.011	0.114	0.302		0.029
**21**	0.303	-0.019	-0.064	0.025	0.348	0.136	-0.069	0.337	-0.044	0.098	0.335	-0.027	-0.019	0.238	-0.037	0.408	0.456	0.071	0.029	
